# Investigating the Factors That Influence Self-Efficacy Among Home Care Workers Providing Dementia Care

**DOI:** 10.1093/geroni/igaa057.1403

**Published:** 2020-12-16

**Authors:** Marie Savundranayagam, Anaya Ahmad, Shalane Basque

**Affiliations:** Western University, London, Ontario, Canada

## Abstract

The self-efficacy beliefs of home care personal support workers (PSWs) play a crucial role in their professional competence and subsequent provision of quality care. Understanding the factors that influence self-efficacy of PSWs is critical to ensuring their job satisfaction and retention, and ultimately improving the quality of care provided to home care clients. Currently, there is a lack of literature exploring the factors influencing self-efficacy among home care PSWs who care for clients with dementia. Accordingly, the purpose of this study was to investigate the sources of self-efficacy for home care PSWs. Conventional content analysis of interviews with 15 home care PSWs yielded six categories of sources influencing self-efficacy: insufficient client information provided by employers, lack of supportive communication by employers, restriction of PSWs’ discretion and autonomy by employers, insufficient practical dementia-specific training, sufficient work experience with clients with dementia, and feedback from family caregivers. These findings call for a multi-pronged approach to enhance the self-efficacy of PSWs. In particular, these findings offer specific areas of improvement for employers on how to best support their PSWs. They also highlight the significant role of dementia-specific education and training for PSWs regardless of their experience in the field. Finally, the findings emphasize the importance of family caregivers in the home care context. Taken together, the study’s findings offer insights on how to best support PSWs and ensure stability in the dementia care workforce.

